# Changes of lung parenchyma density following high dose radiation therapy for thoracic carcinomas – an automated analysis of follow up CT scans

**DOI:** 10.1186/s13014-019-1276-2

**Published:** 2019-04-29

**Authors:** Christina Schröder, Rita Engenhart-Cabillic, Sven Kirschner, Eyck Blank, André Buchali

**Affiliations:** 1 Clinic for Radiotherapy and Radiation Oncology, University Clinic Giessen and Marburg, Marburg, Germany; 2 Clinic for Radiation Oncology, Universitätsspital Zürich, Rämistrasse 100, CH-8091 Zürich, Switzerland; 3 Clinic for Radiotherapy and Radiation Oncology, Ruppiner Kliniken GmbH, Neuruppin, Germany

**Keywords:** Lung, Thoracic neoplasms, Radiation injuries, Pulmonary fibrosis, Lung function

## Abstract

**Background:**

An objective way to qualify the effect of radiotherapy (RT) on lung tissue is the analysis of CT scans after RT. In this analysis we focused on the changes in Hounsfield units (ΔHU) and the correlation with the corresponding radiation dose after RT.

**Methods:**

Pre- and post-RT CT scans were matched and ΔHU was calculated using customized research software. ΔHU was calculated in 5-Gy-intervals and the correlation between ΔHU and the corresponding dose was calculated as well as the regression coefficients. Additionally the mean ΔHU and ΔHU in 5-Gy-intervals were calculated for each tumor entity.

**Results:**

The mean density changes at 12 weeks and 6 months post RT were 28,16 HU and 32,83 HU. The correlation coefficient between radiation dose and ΔHU at 12 weeks and 6 months were 0,166 (*p* = 0,000) and 0,158 (p = 0,000). The resulting regression coefficient were 1439 HU/Gy (p = 0,000) and 1612 HU/Gy (p = 0,000). The individual regression coefficients for each patient range from − 2,23 HU/Gy to 7,46 HU/Gy at 12 weeks and − 0,45 HU/Gy to 10,51 HU/Gy at 6 months. When looking at the three tumor entities individually the highest ΔHU at 12 weeks was seen in patients with SCLC (38,13 HU) and at 6 month in those with esophageal carcinomas (40,98 HU).

**Conclusion:**

For most dose intervals there was an increase of ΔHU with an increased radiation dose. This is reflected by a statistically significant, although low correlation coefficient. The regression coefficients of all patients show large interindividual differences.

## Background

Radiation therapy (RT) of thoracic tumors is commonly associated with toxicities of the surrounding organs at risk. In case of lung or esophageal tumors this usually refers to radiation-induced lung disease (RILD) like radiation pneumonitis (RP) and lung fibrosis [1–11]. Both are common RT induced toxicities that can occur either weeks up to a few months after RT (early side effects, e.g. RP) or up to years after RT (late side effects, e.g. fibrosis). Common symptoms include fever, coughing and shortness of breath. However, not all patients develop clinically apparent symptoms. There is a number of patients with lung tissue changes that are radiologically visible but have no clinical correlate. Also, patients might simply not have yet developed symptoms during the time of the follow up.

An objective way to qualify the effect of RT on lung tissue that includes subclinical affected patients is the analysis of CT scans after RT, e.g. focusing on the changes in Hounsfield units (ΔHU) over time. Although this method might be too complex to implement it into the daily clinical routine, it is a very useful tool if a detailed and objective evaluation is needed. Unfortunately there is no commercially available software for the automated analysis of lung tissue changes. For research purposes custom software has to be developed for both the matching of pre- and post-RT CT scans and the calculation of the lung tissue density changes. As a result there have only been a few trials focusing on lung density changes after RT and among those even less have used an automated analysis or focused on the correlation between density changes and radiation dose [12–21]. Although there are numerous dose constraints regarding severe clinical side effects such as RP, the question remains if there is a correlation between the visible tissue damage and the corresponding radiation dose. Also, how does the lung density after RT change over time? To answer these questions we analyzed CT after treatment by matching the patients’ follow-up CT with the treatment planning CT and calculating the changes in HU over the course of 6 months. Also, we matched the changes in HU to the underlying radiation dose according to the Dose-Volume-Histogram (DVH).

## Methods

### Patient characteristics

Included in this analysis were the CT scans of curatively treatable patients with intrathoracical carcinoma (NSCLC, SCLC, esophageal carcinoma) with a written consent of participation and a Karnofsky index (KI) of at least 70%. Patients with a lung operation in the patient’s medical history, a relevant pleural effusion visible in the planning CT, a forced expiratory volume in 1 s (FEV1) of less than 1 l, the refusal of participation or a KI of less than 70% were excluded. From April 2012 to October 2015 81 patients with thoracic carcinomas received radio-(chemo-)therapy according to intradepartmental standards. The median age of patients was 66 years. Most patients had NSCLC (*n* = 35) or esophageal cancer (n = 35), followed by SCLC (*n* = 11). All patients completed the treatment protocol. Follow up CT scans were performed 12 weeks and 6 months after RT. CT data of 61 patients was available 12 weeks after RT and data of 51 patients 6 months after RT. Further patient characteristics are shown in Table 1.

**Table 1:** Patient characteristics

	*n* (%)
Sex	
male	65 (80,2%)
female	16 (19,8%)
Chemotherapy	
no	23 (28,4%)
yes	58 (71,6%)
Entity (total radiation dose)	
SCLC (60 Gy)	11 (13,6%)
Esophageal CA (66 Gy)	35 (43,2%)
NSCLC (74 Gy)	35 (43,2%)
Smoking history	
never	6 (7,4%)
present	39 (48,1%)
former	34 (42,0%)
unknown	2 (2,5%)
T	
1	3 (3,7%)
2	9 (11,1%)
3	48 (59,3%)
4	21 (25,9%)
N	
0	3 (3,7%)
1	32 (39,5%)
2	15 (18,5%)
3	31 (38,3%)
M	
0	63 (77,8%)
1	18 (22,2%)
Treatment technique	
IMRT	43 (53,1%)
VMAT (rapid arc)	38 (46,9%)
Total	81 (100,0%)

#### Treatment characteristics

NSCLC patients were treated with a total radiation dose of 74 Gy, SCLC patients with 60 Gy and patients with esophageal carcinoma with 66 Gy. Fraction dose was 2 Gy each. All treatment plans had to match intradepartmental dose constraints and were identically standardized using the PTV. Dose constraints for the lung were V20 Gy < 30%, V30 Gy < 20% and V20 Gy < 1000 ml; for the spinal cord a maximum dose (Dmax) of < 47 Gy; for the esophagus a Dmax < 74 Gy and for the heart a mean dose (Dmean) of < 35 Gy, D(33%) < 60 Gy und D(50%) < 45 Gy. All these dose values refer to biological doses. Eligible patients received chemotherapy according to intradepartmental standards.

#### Analysis of follow up CT scans

Follow up CTs were done 12 weeks and 6 months after RT. For all CT scans a Sensation Open CT (Siemens™) with 2 mm slices was used. To calculate the density changes of the lung tissue over time a patient’s follow up CTs had to be matched to the original treatment planning CT. Because of the slightly different positioning and breathing position of a patient at each scan, the deformation of one of the scans was necessary prior to the matching. Since the treatment planning CT was linked to the dose data and structure files the follow up CT had to get deformed. To calculate the differences in lung density (in Hounsfield units) the treatment planning and the follow up scans were automatically subtracted. Because there was a focus on changes in the lung parenchyma, structures with higher density like the tumor itself, other organs, blood vessels etc. were also subtracted from the analyzed volume including a small safety margin. As mentioned before there is no commercial software available so research software had to be customized. This included CERR (Computational Environment for Radiotherapy Research), an open source library for medical research by the US National Institutes of Health and 3D-Slicer, also open source library for medical research by the National Alliance for Medical Image Computing (NA-MIC). The original data sets were imported, analyzed and the calculated ΔHU values were exported into Microsoft Excel and SPSS for further analysis. In this analysis we focused on mean density changes 12 weeks and 6 months post RT. For further analysis the mean density changes were additionally calculated in 5-Gy intervals. The correlation between ΔHU and the corresponding dose was calculated as well as the corresponding regression coefficients. Also the individual regression coefficients were calculated for each patient. Since this analysis was done with patients being treated for 3 different tumor entities (NSCLC, SCLC, esophageal carcinoma) with resulting different total treatment doses, the mean overall density changes and the density changes in 5-Gy intervals were calculated for each tumor entity as well.

#### Statistical analysis

For the comparison of the mean density changes at 12 weeks and 6 months post RT as well as the tumor entity the T-Test was used. Spearman correlation was used to analyze the correlation between the lung density changes and the radiation dose. Regression was done using a linear regression model without a constant. For statistical analysis Microsoft Excel 2008 and SPSS Version 23 were used.

## Results

**Fig. 1:**
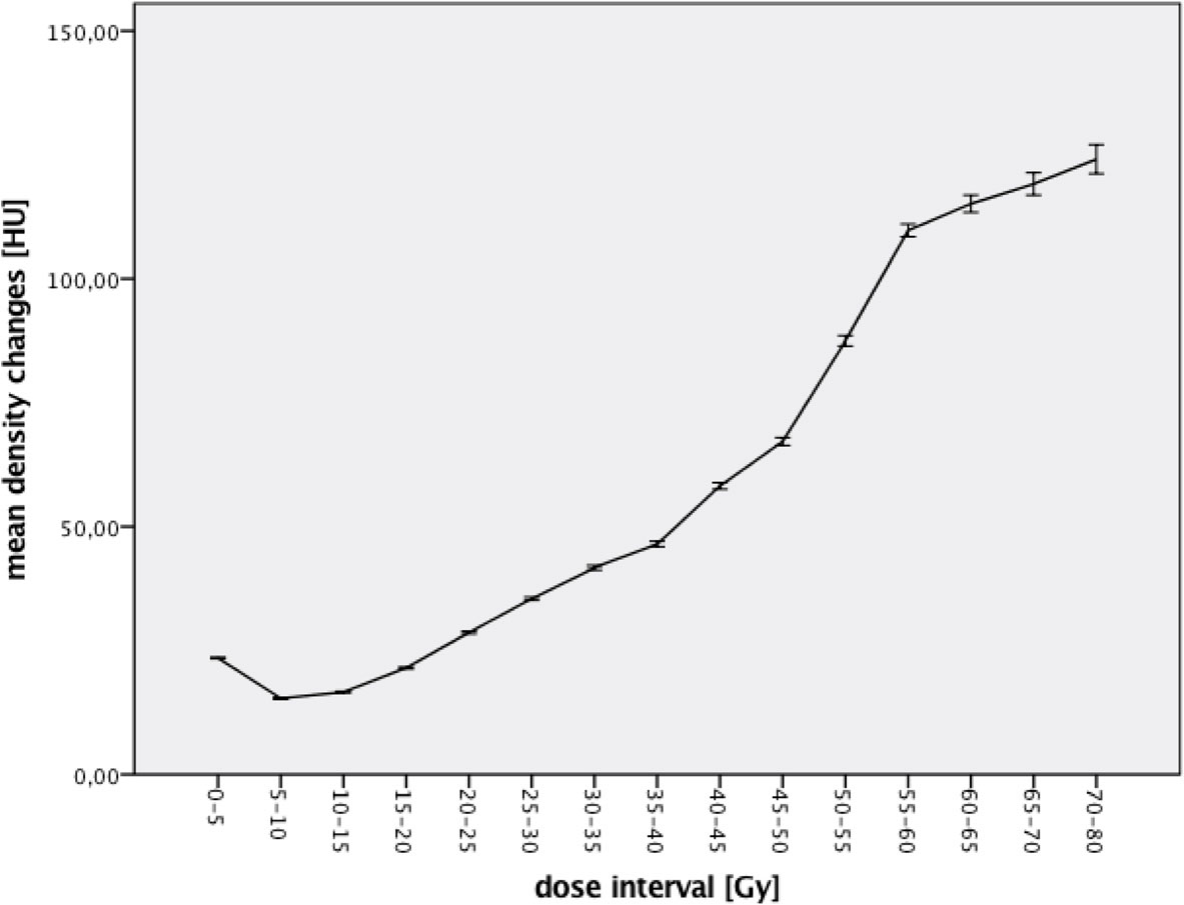
Lung density after RT (with 95% CI)

**Fig. 2:**
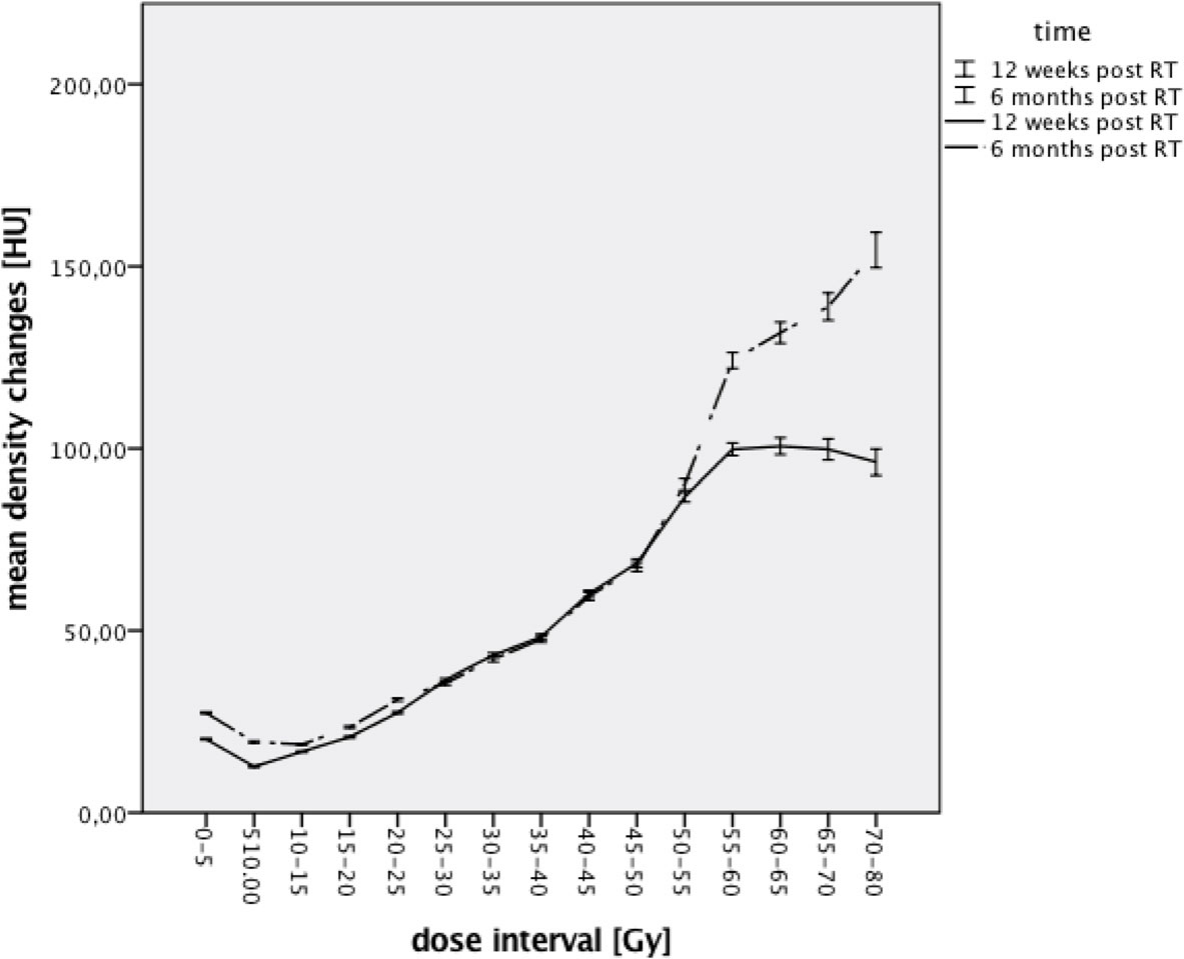
Lung density changes 12 weeks and 6 months after RT (with 95% CI)

Overall, 90% of the values for the density changes of the lung parenchyma range between 0 HU and 300 HU. About 5% of the values were negative, the majority of those between − 100 HU and 0 HU. The mean density changes at 12 weeks post RT were 28,16 HU and 32,83 HU at 6 months post RT. For further analysis the mean density changes were calculated in 5-Gy intervals. Figure 1 shows the mean lung tissue density changes with their corresponding 95% confidence interval (CI) for all data and Fig. 2 shows the curves for 12 weeks and 6 months separately.

**Fig. 3:**
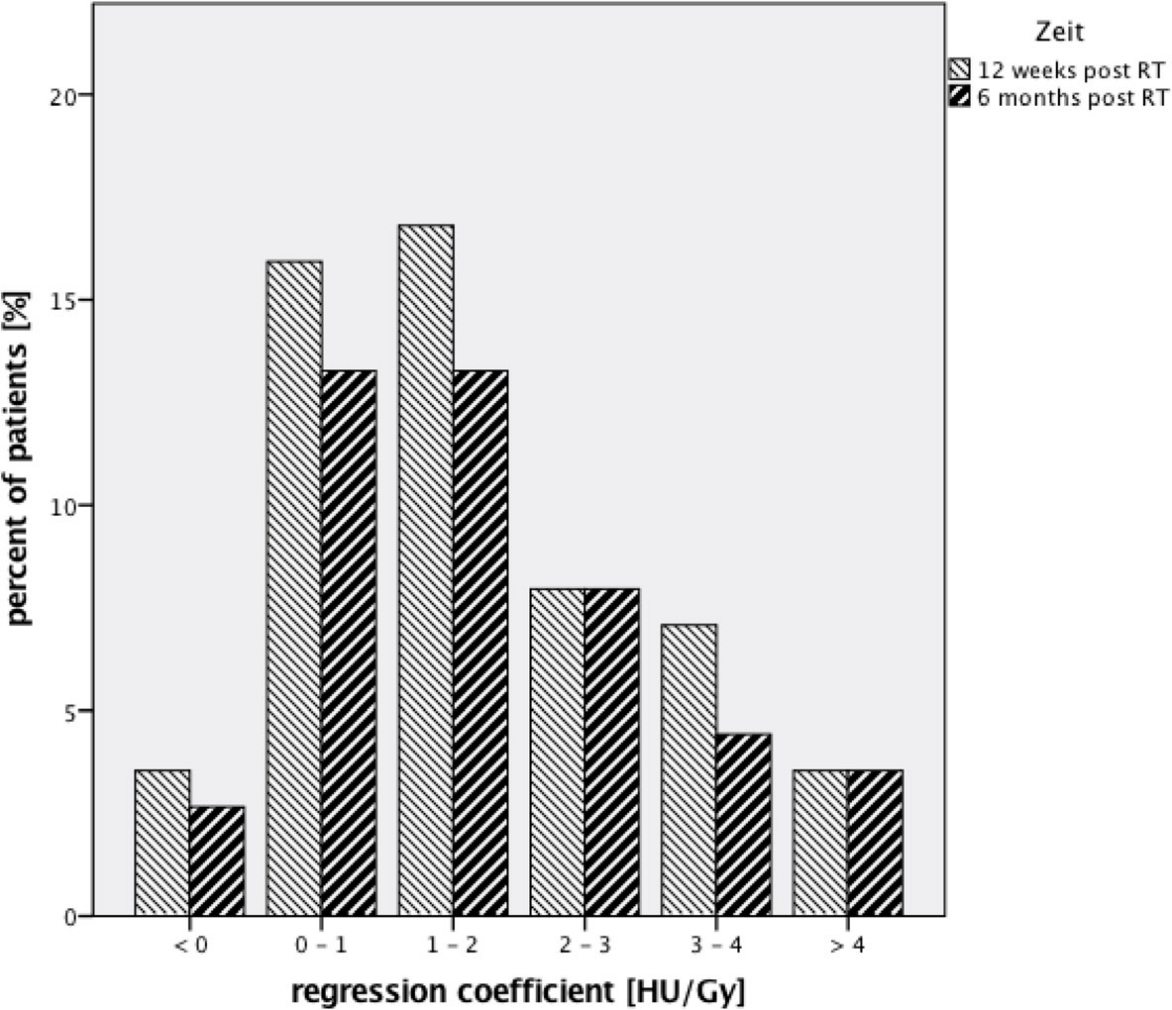
Overview of regression coefficients of patient

The correlation coefficient between radiation dose and density changes of the lung parenchyma for all available values was 0,162 (*p* = 0,000). When looking at 12 weeks and 6 months individually the coefficients were 0,166 (p = 0,000) and 0,158 (p = 0,000). The resulting regression coefficient was 1516 HU/Gy (p = 0,000) for all values and 1439 HU/Gy (p = 0,000) and 1612 HU/Gy (p = 0,000) when looking at 12 weeks and 6 months separately. The individual regression coefficients for each patient range from − 2,23 HU/Gy to 7,46 HU/Gy at 12 weeks and − 0,45 HU/Gy to 10,51 HU/Gy at 6 months. Figure 3 shows the distribution of the regression coefficients of patients.

**Fig. 4:**
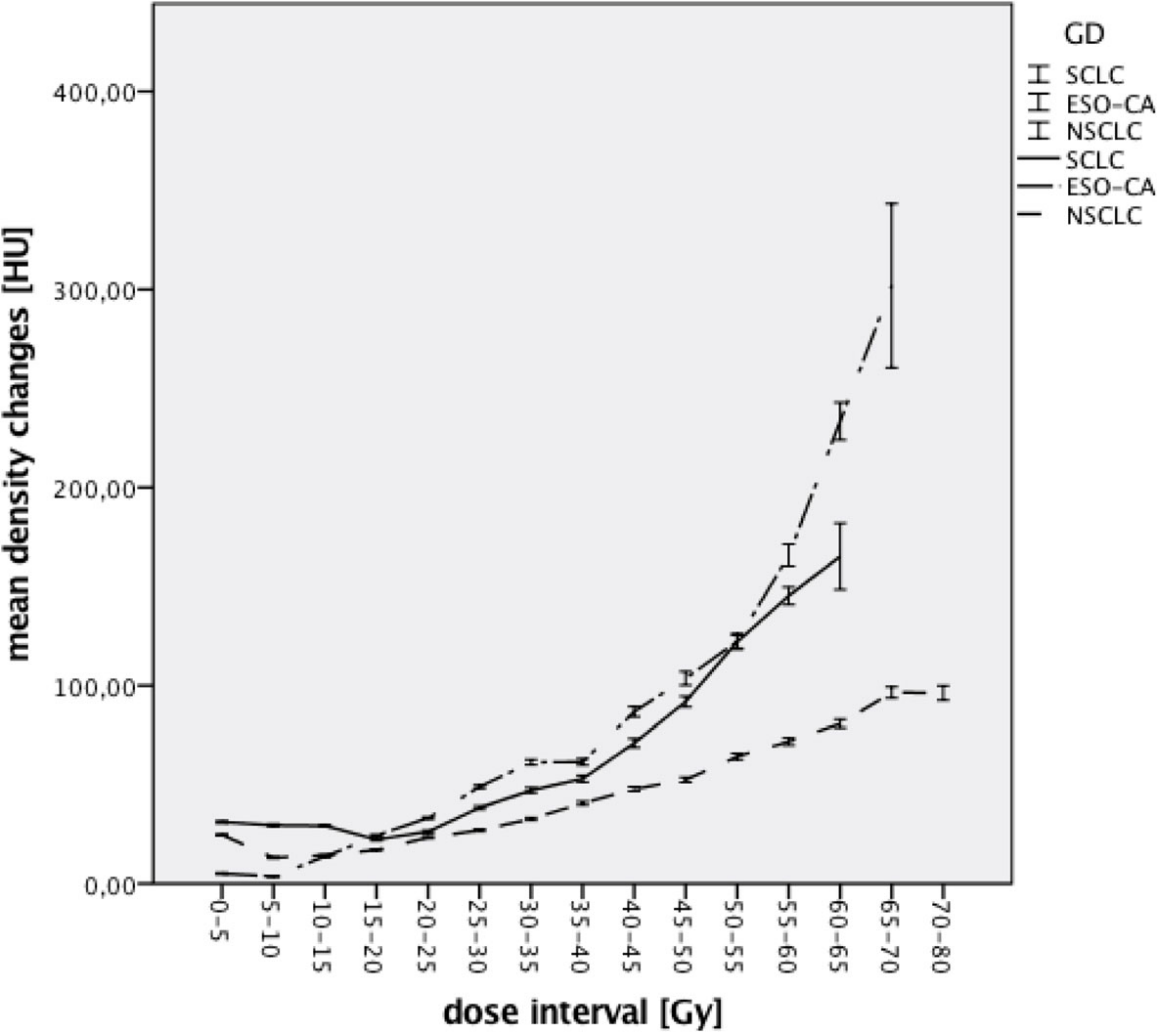
ΔHU 12 weeks after RT for each tumor entity (with 95% CI, *n* = 62)

**Fig. 5:**
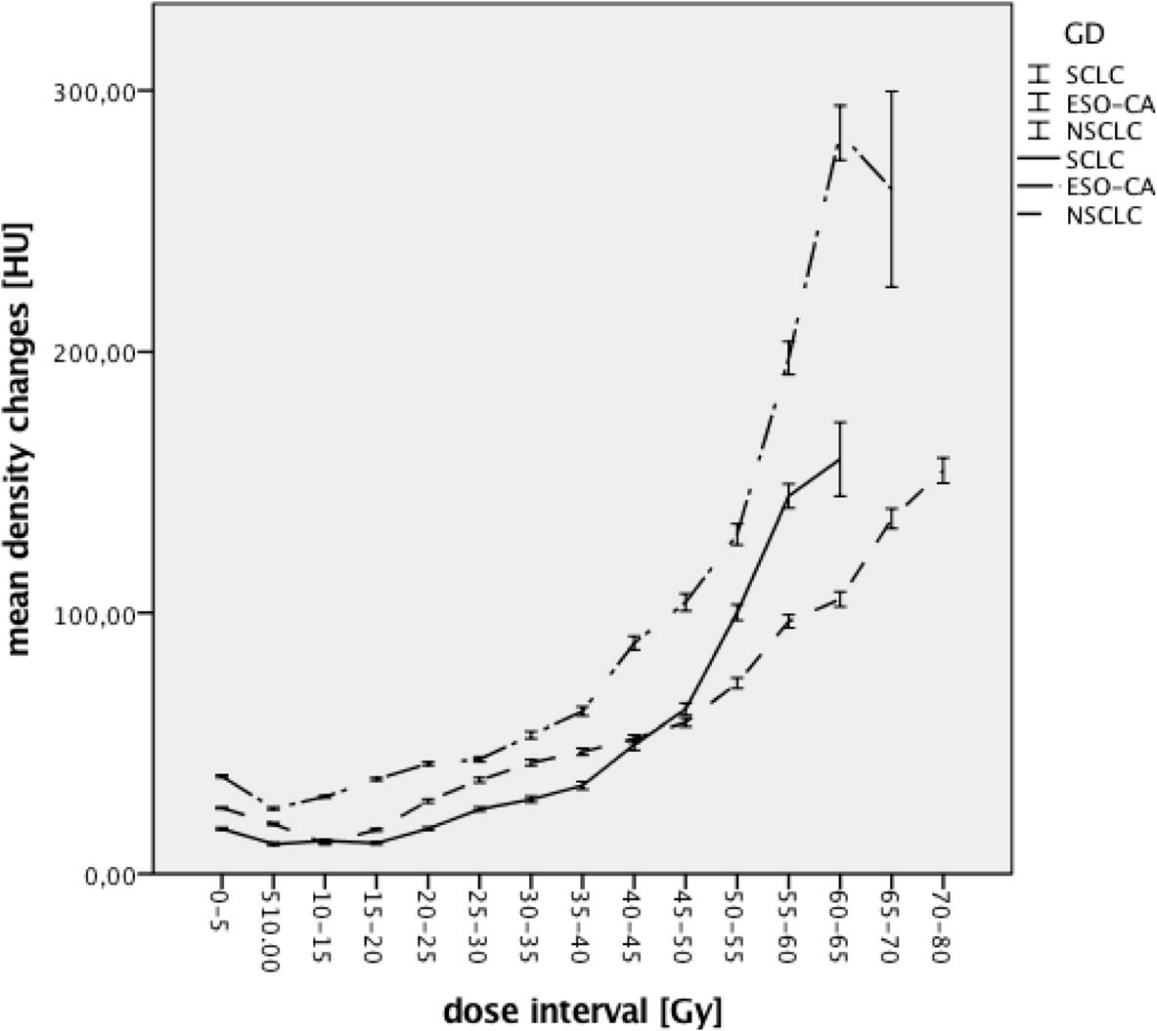
ΔHU 6 months after RT for each tumor entity (with 95% CI, *n* = 51)

When looking at the three tumor entities individually the highest ΔHU at 12 weeks was seen in patients with SCLC (38,13 HU) followed by patients with NSCLC (27,08 HU) and esophageal carcinomas (24,42 HU). At 6 months the patients with the highest ΔHU were those with esophageal carcinomas (40,98 HU) followed by those with NSCLC (31,57 HU) and SCLC (23,10 HU). There was a statistically significant difference for the mean density changes between the tumor entities at 12 weeks as well as 6 months post RT (*p* = 0,000). Figures 4 and 5 show the mean density changes for the tumor entities 12 weeks and 6 months post RT (with 95% CI).

## Discussion

The analysis of post RT CT scans is associated with a lot of challenges deriving from the interindividual differences, the question of background correction and the questionable correlation with clinical symptoms [12–14].

To avoid inaccuracies due to patient positioning and breathing an additional margin was added around the tumor itself and the large intrapulmonary structures like bronchi and vessels before subtraction of the CT scans. A more detailed approach would include repetitive CT scans of a patient, preferably without structural lung damage to determine the background, which is ethically problematic. With the assumption that possible inaccuracies equally lead to a density increase and decrease the original data sets without any background correction were used for this analysis.

Regarding the influence of the radiation dose on the mean density changes there was an increase in lung density for increasing radiation doses for most dose intervals. For a radiation dose of lower than 10 Gy there was an initial decrease in ΔHU. Generally an increase of lung density with increasing radiation dose has been described before [12–16, 22]. Overall the curve is not linear but resembles a sigmoid curve. A sigmoid dose responds curve has been discussed before by Defraene et al. and Bernchou et al. [13, 22]. The initial decrease for low doses might be due to background noise but could also indicate the existence of a threshold for radiation induced density changes. There is not much data on this topic. The data that have been published show a wide range of possible thresholds from under 5–10 Gy to 30–40 Gy [13, 14, 16]. This range is most likely the result of different methods of data analysis but might also be connected to the great interindividual differences described by de Ruysscher et al. [12] and also seen in this analysis. Another hypothesis for the initial decrease is that shrinkage in the high dose areas and a reduced pulmonary function as a result of a fibrosis leads to a compensatory overinflation of lung tissue in the low dose areas. When looking at the density changes over time, the curves for the mean density changes 12 weeks and 6 months post RT overlap between 25 Gy and 50 Gy. For dose values lower than 25 Gy and higher than 50 Gy there is a greater increase in mean density 6 months after RT. The high dose areas are more important for fibrotic changes, which is a late toxicity and according to Krengli et al. has a threshold at about 25 Gy [3, 19, 23, 24]. At 12 weeks the dominant lung toxicity is the RP, which also leads to an increase lung tissue density due to edema. These changes also occur in areas receiving a lower radiation dose [2, 5, 6, 24]. Therefore the density changes for the intermediate dose intervals overlap due to the density changes at 12 weeks as a result of edema and beginning structural changes and at 6 months due to advanced structural changes. At higher doses the fibrotic changes are even greater resulting in a higher mean density as compared to 12 weeks. At doses of lower than 25 Gy there is the problem of background noise and a possible threshold for RT induced density changes. Nevertheless the higher mean density changes 6 months post RT might be the result of more advanced structural changes.

The correlation between lung density changes and radiation dose is statistically significant, although the correlation coefficient is 0,162 and therefore not suggesting a very high level of interaction. Also the overall regression coefficient of 1516 HU/Gy shows that the increase of the mean density with increasing radiation dose is not steep. The individual coefficients range from − 2,23 HU/Gy to 7,46 HU/Gy 12 weeks post RT and − 0,45 HU/Gy to 10,51 HU/Gy 6 months post RT, which shows the underlying interindividual variability. About 75% of patients had a regression coefficient between 0 and 3 at 12 weeks and 6 months suggesting that the majority of patients only show a modest increase in lung density after RT. A similar result has already been published before by de Ruysscher et al. with a mean regression coefficient of 1,8 (SD 2,1 HU/Gy) and 1,4 (SD 1,3 HU/Gy) [12].

There is a statistically significant difference of the mean density changes between the 3 tumor entities. However there is no consistency as to patients with which entity show the largest increase in lung tissue density. 12 weeks post RT the largest mean increase was seen in patients with SCLC, 12 weeks post RT in patients with esophageal carcinomas. This might simply be due to the fact that the total treatment doses are different and therefore the resulting biologically equivalent doses differ as well. Another factor might be that in this cohort patients with NSCLC and SCLC have a pathology of the lung itself and therefore the density changes are not solely the result of RT induced toxicity but also the tumor itself. In general it is questionable if the differences between the tumor entities are not only statistically significant but clinically relevant as well.

## Conclusion

For most dose intervals there is an increase of lung density with an increased radiation dose. Due to possible background noise a definite statement regarding a threshold for RT induced density changes is difficult. The dose-volume relationship is reflected by a statistically significant, although low correlation coefficient. The regression coefficient further describes the relationship between dose and corresponding lung density and shows large interindividual differences. When looking at the mean density changes over time the resulting curves overlap for doses between 25 and 50 Gy, possibly as a result of a density increase due to edema (12 weeks post RT) and structural changes (6 months). Higher mean density changes 6 months post RT are most likely the result of more advanced structural changes, as lung fibrosis typically is a late toxicity. The significant differences between the mean density of patients with SCLC, NSCLC and esophageal carcinomas might simply be the result of additional density changes due to the tumor itself in patients with lung carcinomas.

## Abbreviations

CT: Computed tomography
DVH: Dose-volume-histogram
Gy: Gray
HU: Hounsfield units
KI: Karnofsky index
NSCLC: Non-small cell lung cancer
RILD: Radiation-induced lung disease
RP: Radiation pneumonitis
RT: Radiotherapy
SCLC: Small cell lung cancer
